# Cryptococcal meningitis and immune reconstitution inflammatory syndrome in a pediatric patient with HIV after switching to second line antiretroviral therapy: a case report

**DOI:** 10.1186/s12879-020-4797-2

**Published:** 2020-01-21

**Authors:** Ssegujja Boniface Joseph Otto, Paul E. George, Rebecca Mercedes, Nicolette Nabukeera-Barungi

**Affiliations:** 1 Department of Paediatrics and Child Health, Makerere University College of Health Sciences, P.O. Box 7062, Kampala, Uganda; 2 Baylor College of Medicine and Texas Children’s Hospital, Houston, TX USA

**Keywords:** *Cryptococcus neoformans*, HIV, Immune reconstitution inflammatory syndrome (IRIS), Cryptococcal meningitis (CCM), Antiretroviral therapy

## Abstract

**Background:**

Cryptococcal meningitis (CCM) is a common and deadly disease among HIV-infected patients. Notable about CCM is its association with the immune reconstitution inflammatory syndrome (IRIS). Though it has been posited a switch from first to second-line antiretroviral therapy (ART) can induce CCM IRIS, a case presentation of CCM IRIS has not been published.

**Case presentation:**

A 10-year-old, HIV-infected girl who initially presented with severe headache and new-onset seizures, with cerebrospinal fluid that returned antigen, India Ink, and culture positive for *Cryptococcus neoformans*. Notably, 8 weeks prior to seizures, she had switched from first line to second-line ART (abacavir-lamivudine-efavirenz to zidovudine-lamivudine-lopinavir/ritonavir) due to virologic failure, with a viral load of 224,000 copies/milliliter. At time of seizures and 8 weeks on second-line ART, her viral load had reduced to 262 copies/milliliter.

Her hospital course was prolonged, as she had ongoing headaches and developed bilateral cranial nerve VI palsies despite clearance of *Cryptococcus* from cerebrospinal fluid on antifungal therapy and therapeutic lumbar punctures. However, symptoms stabilized, and she was discharged with oral fluconazole. Cranial nerve palsies resolved 10 weeks post discharge and she has remained disease free.

**Conclusions:**

We describe a case of CCM IRIS in a 10-year-old HIV infected child after changing to second-line ART. This case provides evidence that screening for cryptococcal antigenaemia prior to switch from first-line to second-line ART could be an important measure to prevent cryptococcal disease.

## Background

*Cryptococcus neoformans*, an encapsulated fungal pathogen, is one of the commonest causes of meningitis worldwide [1]. As an opportunistic infection, it has a predilection for disease in the immunosuppressed, notably HIV-infected individuals and most prevalent in sub-Saharan Africa [2]. Despite advances in antiretroviral therapy (ART) efficacy and delivery in sub-Saharan Africa, the burden of cryptococcal disease and specifically cryptococcal meningitis (CCM) has remained high. However, for unclear reasons, CCM is less common in children than adults, with an annual incidence of about 10–50 cases per 100,000 HIV-infected children when compared with 120 cases per 100,000 HIV-infected adults [3]. Case series of cryptococcal disease have shown the peak age to be 5–10 years, though one large series also showed increased incidence in children < 1 year [3–7]. Mortality remains high in children, with reported case fatality rates ranging 20–40% [3, 5].

Notable about CCM is its association with the immune reconstitution inflammatory syndrome (IRIS). IRIS describes a paradoxical worsening of clinical symptoms driven by increased inflammatory processes against an underlying infection in patients with a reversal of immune suppression [8]. Cryptococcal-associated IRIS is particularly common, with studies showing prevalence of 8–50% in HIV-infected patients starting ART, and dangerous, with reported mortalities of 27–83% in Africa [9]. For this reason, the 2018 World Health Organization Guidelines recommend screening for *Cryptococcus* infection in adults and adolescents prior to initiation or re-initiation of ART, and to defer treatment of ART for 4–6 weeks in patients with CCM [10]. Of note, screening for *Cryptococcus* infection is not recommended in children due to the low incidence of cryptococcal disease in this age group and the recommendations make no mention of screening when switching from first to second or third-line ART.

Given that IRIS occurs with rapid immune reversal, it has been posited that ART-associated *Cryptococcus* with IRIS could occur with a switch from first to second-line ART [9]. However, to our knowledge no case report of this so-called unmasking cryptococcal IRIS has been published. Here, we describe a case of CCM IRIS in a 10-year-old HIV infected child after changing to second-line ART.

## Case presentation

A 10-year-old HIV-infected girl who presented to Mulago National Referral Hospital in Kampala, Uganda with a new-onset, generalized tonic-clonic seizure, which resolved with rectal diazepam given in the hospital. The seizure was preceded by a severe frontal headache and subjective fevers for 3 days. Otherwise, she did not have rash, vomiting, diarrhea, night sweats, or weight loss at presentation. There were no known contacts with tuberculosis. On initial exam, she was well appearing, with no abnormalities in vital signs or neurologic examination.

Cerebrospinal fluid (CSF) results showed WBC of 0–1 per high powered field (hpf), red blood cells (RBC) 1–2/hpf, protein 43 mg/dL, glucose 2.5 mmol/L (normal 3.3–4.4). Rapid cryptococcal antigen in CSF and blood were positive. An acid-fast stain and Indian ink stain were positive (++) for yeast cells. An opening pressure was not obtained due to lack of supplies. Two days later, the CSF culture returned positive (++) for *Cryptococcus neoformans*, and she was diagnosed with cryptococcal meningitis. Bacterial meningitis and HIV encephalopathy were the other considerations. CD4 count at the time of presentation was 445 cells/milliliter.

She was diagnosed with HIV at 6 years of age. Since starting ART, she reported poor adherence, leading to treatment failure and switch to second-line therapy (ABC-3TC-EFV➔AZT-3TC-LPV/r) 8 weeks prior to the onset of her current symptoms; viral load at that time was 224,000 copies/milliliter. Her mother reported excellent adherence, with no missed doses, since starting second-line line ART. Her most recent CD4 count was from 3 years prior at 366 cells/milliliter. There were no recorded cryptococcal antigen tests from the previous year.

**Fig. 1:**
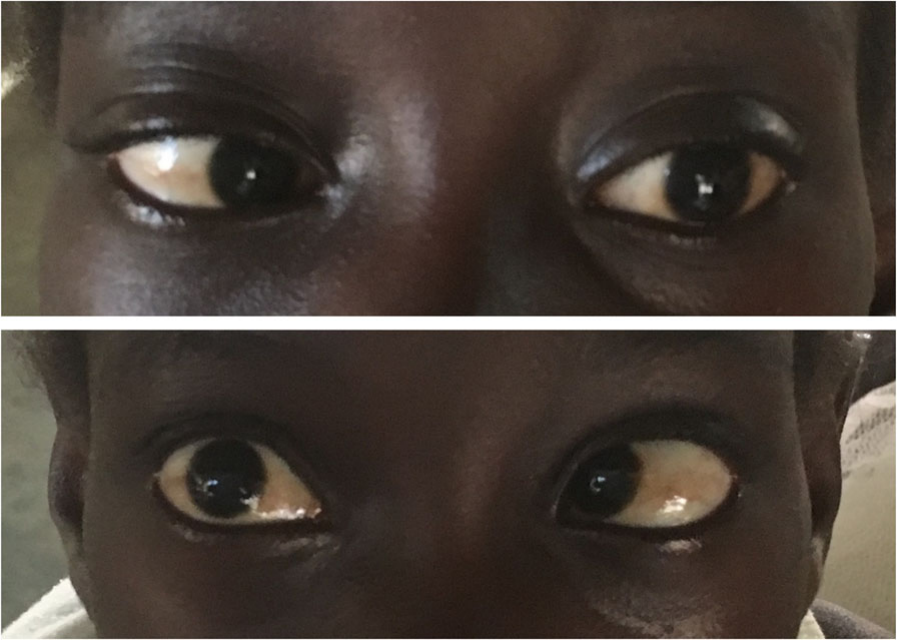
Top Panel: Instructed to look left →. Bottom Panel: Instructed to look right ←

On admission, she was started on amphotericin B deoxycholate (0.8 mg/kg/day) and high dose oral fluconazole (12 mg/kg/day), as flucytosine is not readily available. Given ongoing neurologic symptoms (headache, vomiting), several therapeutic lumbar punctures were performed throughout admission (day 2 of hospitalization 8 mL was drained, day 6, 10 mL, day 10, 15 mL and day 18, 12 mL) with transient improvement in symptoms. Despite this, on day 16 of hospitalization she developed bilateral cranial nerve VI palsy (see Fig. 1). CSF analysis showed a negative India ink stain and culture; CSF cell counts were not obtained due to a shortage of reagents. A CT head with/without contrast from day 17 showed mild parenchymal edema of left parietal lobe, otherwise normal. HIV viral load obtained at that time was 262 copies/milliliter. Given persistent symptoms, her induction therapy of amphotericin B and high dose fluconazole was increased from 14 to 21 days. She did not receive other adjuvant therapy such as glucocorticoids.

At discharge on day 21, she remained with bilateral cranial nerve VI palsy, but otherwise asymptomatic and seizure-free. Her cranial nerve defects resolved within 10 weeks of discharge and she has remained on oral fluconazole. Six months after discharge, she remains without seizures, cranial nerve palsies, or other sequala of CCM.

This case report received IRB approval. We have obtained written consent from the primary caregiver of the child (the mother) and written assent from the child to publish the case report and associated images.

## Discussion

This case highlights the occurrence of cryptococcal IRIS in the form of CCM in a pediatric, HIV-infected patient. This is the first case report of IRIS, a common sequalae of cryptococcal disease upon initiation of ART, described following switch from first to second-line ART.

In this case, the patient developed meningoencephalitic features including fever, headache, seizures and cranial nerve VI palsy. The most common symptoms seen in CCM are headache, fever, nausea, vomiting, dizziness, irritability and somnolence [6, 11]. However, it may be virtually asymptomatic apart from a mild headache [6]. Unlike in adults, meningeal signs and an altered state of consciousness can be absent in HIV-positive children with CCM [12, 13]. For example, in a case report from South Africa involving seven pediatric patients with HIV and CCM, three patients presented with fever and without other localizing symptoms [14].

Cryptococcal IRIS presents as a clinical worsening or new presentation of cryptococcal disease after rapid reversal of immunodeficiency [15]. Cryptococcal IRIS is thought to be triggered by recovery of immune responses to cryptococcal species, resulting in exaggerated host inflammatory responses. In patients with HIV, the syndrome is driven by immune reconstitution due to ART. The syndrome has also been known to occur after solid organ transplantation (estimated incidence 4.8%) [16] and in pregnancy [17]. Notably, in our patient, the CSF indices were not associated with an exaggerated inflammatory response. Previous studies have shown that HIV-infected children with CCM, unlike adults, are more likely to present with normal CSF indices, namely WBC < 6 cells/hpf [13]*.* Studies have also shown that the cytokine response in CCM IRIS is more robust in the peripheral blood than in the CSF [18].

However, per Haddow et al. [9], one clinical definition of an exaggerated inflammatory response is “meningitis … with opening pressure >20 that is refractory to therapy.” The increased opening pressure in cryptococcal meningitis is secondary to decreased reabsorption of CSF due to blockage by the cryptococcal capsule, indicating high burden of disease [19]. Presence of cryptococcal antigen has also been shown to inhibit leukocyte migration possibly accounting for the low WBC count despite relatively high CD4 count in this patient. The persistently present cryptococcus is what leads to the unregulated immune response as the CD4 recovers but may not particularly be reflected at the site of infection [20]. Although we were unable to obtain opening pressures, we presumed our patient remained with high intracranial pressures despite anti-fungal therapy given the persistent headaches, which were temporarily relieved with therapeutic lumbar punctures, and cranial nerve palsies she developed on day 16.

Two distinct modes of presentation of cryptococcal IRIS are recognized, paradoxical and ART-associated cryptococcal IRIS. Paradoxical cryptococcal IRIS presents as a worsening of disease or as a recurrent disease in the same or new anatomical sites, despite microbiological evidence of effective antifungal treatment. It occurs in up to one third of patients with cryptococcosis diagnosed before the initiation of ART [21, 22]. The patient in our case however had no evidence of ongoing cryptococcal disease prior to her 3 days of headaches, seizure, and subsequent diagnosis and treatment. A high index of suspicion is required for early diagnosis and treatment because cryptococcal meningitis IRIS sometimes does not present with overt clinical signs [13]. In our patient, her diagnosis was based on the proposed case definition and clinical checklist criteria from Haddow et al. [9] (Table 1).

**Table 1:** Proposed case definitions for antiretroviral-therapy-associated cryptococcosis and unmasking cryptococcal immune reconstitution inflammatory syndrome

Antiretroviral therapy-associated cryptococcosis 1. Patient taking antiretroviral therapy (ART) 2. No recognized cryptococcal disease at ART initiation 3. Clinical disease worsening caused by cryptococcosis occurs after initiation, re-introduction, or regimen switch after previous failure 4. Cryptococcal infection characterized by meningitis, CNS complications, skin or soft-tissue lesions, lymphadenopathy, lung disease, or disseminated disease	
Unmasking cryptococcal IRIS 1. Criteria for ART-associated cryptococcosis are met 2. Unusual, exaggerated, or heightened inflammatory manifestations 3. Event occurs early after ART initiation (typically, within 3 months) 4. Failure of ART excluded if possible (eg, ≥1.0 log10 copies/mL decrease in HIV-1 viral load by 8 weeks treatment)	

Above proposed definitions taken from Haddow et al. Cryptococcal immune reconstitution inflammatory syndrome in HIV-1-infected individuals: proposed clinical case definitions. Lancet Infect Dis 2010;10(11):791–802

The case is therefore more consistent with the later mode of presentation (ART-associated cryptococcal IRIS) in which new onset cryptococcosis occurs after ART is started in patients whom cryptococcosis was not recognized before treatment, occurring in up to 1% of patients [23]. Due to the difficulties in the differentiation between IRIS-associated disease (unmasking cryptococcal IRIS) and progression of untreated occult cryptococcosis in the context of persisting immunodeficiency, the term ‘ART-associated cryptococcosis’ has been preferred for both [9]. The diagnosis of IRIS in resource-limited settings, our case included, has been made possible by adopting the clinical criteria checklist approach set by the International Network for the Study of HIV-associated IRIS since there is no definitive diagnostic test.

The onset of our patient’s seizures was rapid, occurring just 3 days from the time of first complaint of fever and headache. This is consistent with ART-associated CCM which is often characterized by rapid development of severe illness, developing over a few days from the onset of symptoms [24], compared with the 1–2 week subacute course typically seen with CCM in patients not receiving ART [21]. From individually reported times of symptom onset, the median time after the start of ART was 9 weeks (IQR 2–26 weeks) in patients with paradoxical cryptococcal IRIS and 4 weeks (IQR 2–10 weeks) in those with ART-associated cryptococcosis [9]. The patient in this case report developed symptoms of CCM 8 weeks after switching from first to second-line ART.

Interestingly, our patient’s CD4 of 445 cells/milliliter was higher than typically recorded for patients with IRIS. However, this CD4 count was taken at time of diagnosis of CCM, 8 weeks after initiation of second-line ART. As such, her CD4 count was likely significantly lower when starting second-line, as previous studies have shown rises of CD4 counts of 50–150 cells/milliliter within the first 6–12 weeks after starting protease-inhibitor second-line ART [25]. Further, IRIS is most likely to occur in the setting of a rapidly rising CD4 count [26], and weeks 6–18 have been shown to be weeks when CD4 counts rise the fastest [27]. Thus, while her CD4 count at diagnosis of CCM was unusually high, her overall clinical trajectory is typical for CCM IRIS.

ART-associated cryptococcosis is caused by either restoration of a *Cryptococcus* specific immune response (i.e., unmasking cryptococcal IRIS) or by persistent immunodeficiency while receiving ART [9]. Although the patient in this case report had evidence of ART failure with persistence of immunodeficiency prior to switching ART, there was no evidence of CCM. This case therefore correlates more with unmasking ART-associated cryptococcal IRIS, likely in the setting of immune restoration that occurred following the switch of ART, as evidenced by the drop in the viral load from 224,000 copies/ milliliter to 262 copies/ milliliter within a period of 3 months. While not required to make a diagnosis, a specific threshold for a reduction in viral load of greater than 1.0Log10 copies/milliliter at the time of clinical event supports the diagnosis of IRIS [28]. In the case of our patient, the drop in the viral load following switch of ART was 2.9 Log10, significantly higher than 1.0 Log10 threshold. The significant drop in viral load allows for immune reconstitution to occur and therefore is used a surrogate marker of immune reconstitution. The best time to do viral load assays or the best threshold value or values have however not been experimentally validated [9]. The clinical distinction of the two causes of ART-associated cryptococcal IRIS is important because clinical management could be affected by this distinction and also because both causes are important in regions of high cryptococcal prevalence, our setting inclusive [9].

Subclinical antigenaemia is the overwhelming risk factor for ART-associated cryptococcosis, with the incidence nearly 33% in individuals with subclinical cryptococcal antigenaemia without pre-emptive therapy with fluconazole [29]. In our patient, it is possible that she had subclinical antigenaemia; however, no screening test was done prior to switching of ART. In addition, the World Health Organization guideline does not mention screening for children, only for adults and adolescents. The guideline recommends screening for cryptococcal antigen followed by pre-emptive antifungal therapy among cryptococcal antigen–positive people to prevent the development of invasive cryptococcal disease for adults and adolescents living with HIV who have a CD4 cell count < 100 cells/mm^3^ [10]. Furthermore, both the WHO and Ugandan national guidelines only recommends screening in patients prior to initiation or re-initiation of ART; it makes no mention of screening prior to switch from first to second or second to third line therapy. As such, our patient did not undergo cryptococcal screening prior to switch from first to second line therapy. Given that IRIS occurs with rapid immune reversal, it has been posited that ART-associated *Cryptococcus* with IRIS could occur with a switch from first to second-line ART [9]. However, to our knowledge no case of this so-called unmasking cryptococcal IRIS following switch from first to second-line therapy has been reported, either in adult or pediatric literature.

In conclusion, cryptococcal IRIS is thought to be triggered by recovery of immune responses to cryptococcal species, resulting in exaggerated host inflammatory responses. In patients with HIV, the syndrome is due to the immune reconstitution driven by effective ART, which could plausibly occur with a switch from first to second line ART. This case therefore highlights the need for future studies on cryptococcal antigenaemia in children not only at initiation of ART but also prior to switching between lines of therapy in children, adolescents, and adults, as such screening could have important clinical implications for the prevention of cryptococcal disease.

## Data Availability

Not applicable.

## Abbreviations

ART: Antiretroviral therapy
CCM: Cryptococcal meningitis
CSF: Cerebrospinal fluid
IRIS: Immune reconstitution inflammatory syndrome
